# Dietary supplementation with mulberry leaf flavonoids and carnosic acid complex enhances the growth performance and antioxidant capacity via regulating the p38 MAPK/Nrf2 pathway

**DOI:** 10.3389/fnut.2024.1428577

**Published:** 2024-07-30

**Authors:** Chunming Liu, Hui Huang, Yulian Chen, Yingjun Zhou, Tiantian Meng, Bihui Tan, Wenxiang He, Xiaoqin Fu, Dingfu Xiao

**Affiliations:** ^1^College of Animal Science and Technology, Hunan Agricultural University, Changsha, China; ^2^College of Xiangya Pharmaceutical Sciences, Central South University, Changsha, China; ^3^Geneham Pharmaceutical Co., Ltd., Changsha, China; ^4^Yuelushan Laboratory, Changsha, China

**Keywords:** mulberry leaf flavonoids, carnosic acid, broiler, growth performance, antioxidant

## Abstract

**Introduction:**

This study aimed to investigate the regulatory effects of mulberry leaf flavonoids and carnosic acid complex (MCC) on the growth performance, intestinal morphology, antioxidant, and p38 MAPK/Nrf2 pathway in broilers.

**Methods:**

A total of 256 healthy 8-day-old female yellow-feathered broilers were randomly divided into 4 equal groups: a control group (CON) fed a basal diet, an antibiotic group (CTC) supplemented with 50 mg/kg chlortetracycline, and two experimental groups (MCC75, MCC150) fed basal diets with 75 mg/kg and 150 mg/kg of MCC, respectively. The experiment lasted for 56 days, with days 1–28 designated as the initial phase and days 29–56 as the growth phase.

**Results:**

The results on the growth performance showed that diets supplemented with MCC and CTC decreased the feed-to-gain ratio (F/G), diarrhea rate, and death rate, while significantly increasing the average daily weight gain (ADG) (*p* < 0.05). Specifically, the MCC150 group enhanced intestinal health, indicated by reduced crypt depth and increased villus height-to-crypt depth ratio (V/C) as well as amylase activity in the jejunum. Both the MCC and CTC groups exhibited increased villus height and V/C ratio in the ileal (*p* < 0.05). Additionally, all treated groups showed elevated serum total antioxidant capacity (T-AOC), and significant increases in catalase (CAT) and glutathione peroxidase (GSH-Px) activities were observed in both the MCC150 and CTC groups. Molecular analysis revealed an upregulation of the jejunal mRNA expression levels of PGC-1α, Nrf2, and Keap1 in the MCC and CTC groups, as well as an upregulation of ileum mRNA expression levels of P38, PGC-1α, Nrf2, and Keap1 in the MCC150 group, suggesting activation of the p38-MAPK/Nrf2 pathway.

**Discussion:**

These findings indicate that dietary supplementation with MCC, particularly at a dosage of 150 mg/kg, may serve as a viable antibiotic alternative, enhancing growth performance, intestinal health, and antioxidant capacity in broilers by regulating the p38-MAPK/Nrf2 pathway.

## 1 Introduction

The expansion of chicken farming is associated with substantial challenges, including environmental stressors, prevalent diseases, and substandard feeding practices, which significantly compromise the immune health and growth performance of broilers (1). While antibiotics have been employed to enhance disease resistance and boost production metrics, their use has been marred by considerable drawbacks, notably the specter of antibiotic residues persisting in poultry products (2). Antimicrobial resistance has escalated into a critical global health emergency, spurred by the overutilization and incorrect application of antibiotics within the realm of animal husbandry, which catalyzes the emergence of resistant microbial strains (3). Specifically, the poultry industry has been implicated as a major source of this problem, wherein the routine use of antibiotics for growth promotion has elicited heightened concern over potential impacts on human health and environmental safety (4). Therefore, it is urgent to explore alternative strategies to promote poultry growth and disease prevention. Plant extracts, which have been shown to possess antimicrobial, antioxidant, and immune-stimulating properties, emerge as a promising avenue for diminishing the reliance on antibiotics in poultry farming (5, 6).

Mulberry leaf flavonoids are one of the significant active components of mulberry plants, mainly including quercetin, kaempferol, rutin, morin and its derivatives (1). Extensive research indicates that these flavonoids can effectively improve the antioxidant properties and oxidative stress resilience of broilers, alongside bolstering their immunity and disease resistance (7, 8). Moreover, these compounds have been found to facilitate the growth and development of broilers, suggesting their promising utility in broiler production practices (1). Carnosic acid is a phenolic diterpenoid primarily extracted from rosemary and other Lamiaceae plants, and its content in air-dried rosemary leaves can range from 3 to 10% (9). There are currently few studies on the application of carnosic acid, which is a natural fat-soluble antioxidant with demonstrated antibacterial, anti-inflammatory, and antioxidant effects (9, 10). Although these two natural chemicals have been the subject of extensive research in recent years due to their health benefits, their complexes have received comparatively little attention. Based on the extensive research on these two natural substances, we hypothesize that their complex has growth-promoting and antioxidant effects on livestock and poultry, potentially serving as a substitute for antibiotics. Therefore, the major objectives of this study were to investigate the effects of dietary supplementation with mulberry leaf flavonoids and carnosic acid complexes (MCC) on broiler performance, nutrient digestibility, intestinal digestive enzymes, intestinal morphology, and antioxidant properties, to evaluate the potential of MCC as a green feed additive.

## 2 Materials and methods

### 2.1 Animal ethics statement

To ensure animal welfare, all experiments and methods are designed to minimize animal suffering. All experiments and sample collection procedures were performed according to the Chinese guidelines for animal welfare and were approved by the Institutional Animal Care and Use Committee of Hunan Agricultural University (Permit Number: CACAHU 2020-0821).

### 2.2 Animals and experimental treatments

A total of 256 healthy 8-day-old female yellow-feathered broilers, with an average initial body weight of 101.0 ± 2.0 g, were randomly divided into four groups. Each group had 8 replicates of 8 birds each. The four groups were denoted as the CON group (basal diet), the CTC group (basal diet with 50 mg/kg chlortetracycline), the MCC75 group (basal diet supplemented with 75 mg/kg MCC), and the MCC150 group (basal diet supplemented with 150 mg/kg MCC). The addition dosage of MCC was determined based on the preliminary experiments of our research team. The experiment included a 7-day pretest period and a subsequent 56-day trial period. According to the standard nutritional requirement of broilers (Agricultural industry standard of the people’s Republic of China—chicken breeding standard NY/T33-2004), the basic diet formula of the formal trial period was divided into two periods (d 1–28 and d 29–56), and its nutrient profile is shown in Table 1.

**Table 1:** Ingredients and nutrients composition of the basal experimental diet (air-dry basis, %).

Ingredients	1–28 days	29–56 days	Nutrient level^b^	1–28 days	29–56 days
Corn	62.20	67.50	ME (MJ/kg)	12.40	12.54
Soybean meal	28.00	28.00	CP	20.49	18.80
Puffed soybeans	6.00	0.00	Lysine	1.13	1.00
Soybean oil	0.10	1.20	Methionine	0.46	0.40
DL-Methionine	0.10	0.10	Cystine + Methionine	0.83	0.74
Dicalcium phosphate	1.50	1.30	Ca	1.00	0.90
Stone powder	1.20	1.10	AP	0.45	0.40
Choline	0.10	0.00			
NaCl	0.30	0.30			
Premix^a^	0.50	0.50			
Total	100.00	100.00			

a Provided per kilogram of diet: 50 mg of Cu; 50 mg of Fe; 50 mg of Mn; 60 mg of Zn; 1 mg of I; 0.5 mg of Se; 50,000 IU of vitamin A; 15,000 IU of vitamin D3; 130 IU of vitamin E; 10 mg of vitamin K3; 20 mg of vitamin B1; 0.5 mg of vitamin B2; 0.5 mg of vitamin B6; 75 mg of vitamin B12; 0.4 mg of biotin; 30 mg of pantothenic acid; 6 mg of folic acid; 160 mg of nicotinic acid.

b Calculated values.

The MCC used in the experiment was sourced from Hunan Geneham Pharmaceutical Co., Ltd. It consisted primarily of 25% mulberry leaf flavone, 25% carnosic acid, and the remaining components served as carriers. Of which, mulberry leaf flavonoids were obtained by dissolving mulberry leaves in water, followed by two rounds of reflux, and subsequently concentrating and spray drying the filtrate. Carnosic acid was extracted from *Salvia miltiorrhiza* using alcohol, followed by high-pressure filtration and spray drying.

The experiment was conducted at the breeding base of Hunan Agricultural University. Prior to the test, the floor walls of the chicken house and the chicken cage underwent a cleaning, disinfection, and ventilation process for 3 days. The test chickens were raised in four-layer fully enclosed chicken cages (60 cm width × 120 cm length × 50 cm height) with artificial lighting throughout the test. The humidity was controlled at 50–70%, and the temperature was maintained at 33–35°C from d 1–7 and at 27–31°C from d 8–14, gradually reducing to approximately 22°C until d 28. All birds were fed twice a day at 08:00 h and 16:00 h. Water and feed (crumbled) were provided *ad libitum*, the chicken house was regularly cleaned, and immunizations were administered as per standard protocols.

### 2.3 Sample collection

At the end of the trial, one broiler close to the average weight of each replicate was chosen, weighed, and data were collected (8 birds per group, respectively). Subsequently, blood samples from the jugular vein were collected in 10 mL centrifuge tubes, centrifuged at 3,500 r/min for 10 min at 4°C, and the obtained serum samples were stored at −20°C. The birds were euthanized by cervical dislocation. The spleen, thymus, and bursa of Fabricius were removed and weighed. The digesta from the middle jejunum was transferred to a 1.5 mL centrifuge tube, temporarily stored in liquid nitrogen, and then stored at −80°C. Additionally, samples for morphological analysis were collected from the middle jejunum and middle ileum (1–2 cm). The mucosa of jejunum and ileum was collected, rapidly frozen with liquid nitrogen, and stored at −80°C.

### 2.4 Growth performance

The broilers were weighed on the 0th, 28th, and 56th days of the experiment, and the feed intake was recorded during the experiment to calculate the average daily feed intake (ADFI), average daily gain (ADG), feed conversion ratio (F/G), diarrhea rate, and death rate of broilers in the early, late, and overall stages. The diarrhea rate was calculated as follows: Diarrhea rate (%) = number of diarrhea broilers/ (total number of broilers × total experimental days) × 100. Diarrhea was defined as watery manure with an irregular shape.

### 2.5 Immune organ index

The immune organ index (*n* = 8) was calculated by dividing the fresh weight (g) of the immune organs (thymus, spleen and bursa of Fabricius) by the pre-slaughter weight (g) of the chickens (6).

$\begin{array}{l} \mathrm{Immune}\;\mathrm{organ}\;\mathrm{indexes}\;\left(\%\right)=100\times \mathrm{Immune}\;\mathrm{organ}\;\mathrm{weight}\;\left(g\right)\\ \;/\mathrm{body}\;\mathrm{weight}\;\left(g\right) \end{array}$ .

### 2.6 Apparent digestibility of nutrients

During the final 7 days of the experiment (d 50–56), 0.3% titanium dioxide (TiO_2_) was added to the diet as an exogenous indicator, and feed samples were randomly collected from each group and stored for testing. Fecal samples were collected on the last 4 days of the test period (d 53–56), and approximately 50 g of representative fecal samples were collected daily in the fecal pan under each cage using a five-point method to remove debris such as feathers and dander in the feces, and 10 mL of 10% dilute sulfuric acid was added to each 100 g of feces for nitrogen fixation and stored in a freezer at 20°C. The fecal samples were then properly combined, primary dried for approximately 6 h at 65°C, and weighed before being kept for testing. The crude extract content of ether in feces and feed was determined by the Soxhlet extraction method, the crude ash content of feces and feed was determined by high temperature ignition at 550°C, the crude protein content was determined by the Kjeldahl method, and the crude fiber content was determined by a semi-automatic fiber detector. The apparent digestibility of nutrients in the diet was calculated according to the following formula (11):

$\mathrm{AD}\;\left(\%\right)=\left[1-\left(G1\times F2\right)/\left(G2\times F1\right)\right]\times 100$ .

AD, apparent digestibility of nutrients in the diet; G1, content of titanium in the diet; F1, content of a nutrient in the diet; G2, content of titanium in feces; F2, content of a nutrient in feces.

### 2.7 Intestinal digestive enzyme activity

The amylase (AMY, Kit Serial No: C016), lipase (LIP, Kit Serial No: A054), and trypsin (TP, Kit Serial No: A080) levels in jejunal contents (*n* = 8) were determined following the protocols of commercial kits (Nanjing Jiancheng Bioengineering Institute, Nanjing, China), according to the manufacturer’s instructions.

### 2.8 Morphological structure of intestinal tract

Briefly, intestinal samples were dehydrated and embedded in paraffin (Thermo Fisher Scientific, Kalamazoo, MI, United States), and then sectioned into 4-μm thick histological slices for hematoxylin and eosin staining (HE). Representative fields were chosen for photography from a large number of randomly selected non-continuous fields. The ratio of villus height to crypt depth was calculated by measuring intestinal villus height and crypt depth with IMAGEEX, an image analysis program included in the YLE-21DY microscopic imaging system (Leica, Germany).

### 2.9 Serum antioxidant

The activities of total superoxide dismutase (T-SOD, Kit Serial No: A001), glutathione peroxidase (GSH-Px, Kit Serial No: A005), catalase (CAT, Kit Serial No: A007), total antioxidative capacity (T-AOC, Kit Serial No: A015), and the content of malondialdehyde (MDA, Kit Serial No: A003) were assayed (*n* = 8) using colorimetric methods with a Microplate Reader (Infinite M200 PRO, TECAN, Switzerland). The assays were conducted using the commercial kits purchased from Nanjing Jiancheng Biotechnology Co., Ltd. (Nanjing, China) and following their corresponding procedures.

### 2.10 Expression of antioxidation-related gene in the intestinal p38-MAPK/Nrf2 pathway

Total RNA (*n* = 8) was extracted from the jejunum and ileum mucosa by using the Trizol method (R401-01, Vazyme, Nanjing, China), and then reverse transcription and real-time quantitative PCR were performed using the reverse transcription kit (R223-01, Vazyme, Nanjing, China) and fluorescence quantitative kit (Q711-02, Vazyme, Nanjing, China), respectively. The quality and quantity of extracted RNA were determined using a Nanodrop Spectrophotometers (IMPLEN, CA, United States) and a Qubit Fluorometer (Life Technologies, CA, United States). The primers for the target gene were synthesized by Tsingke Biotechnology Co., Ltd. (Table 2). Real-time PCR analysis of the gene expression was performed using SYBR Green (Thermo Fisher Scientific, MA) on an ABI 6 flex real-time PCR instrument (Thermo Fisher Scientific). The reaction conditions were as follows: 50°C for 2 min, 95°C for 10 min; 40 cycles of 95°C for 15 s, 60°C for 1 min. Melt curve analysis was performed to confirm the PCR amplification specificity (12). The target gene relative expression was calculated according to the 2^−ΔΔCt^ method and the housekeeping gene *β-actin* was chosen as an internal reference gene.

**Table 2:** Sequence of primers for real-time PCR.

Target gene	Accession no.	Nucleotide sequence of primers (5′-3′)	Product size (bp)
β-actin	NM_205518.2	F: ATGATGATATTGCTGCGCTCGT	139
		R: CCCATACCAACCATCACACCCT	
JNK	NM_205095.1	F: TGACCGAGTGAGGAGACGAT	211
		R: ACTGTATCGAACGCAGCACA	
TNF-ɑ	NM_204267	F: TGTGTATGTGCAGCAACCCGTAGT	229
		R: GGCATTGCAATTTGGACAGAAGT	
IL-6	NM_204628.1	F: AAATCCCTCCTCGCCAATCT	106
		R: CCCTCACGGTCTTCTCCATAAA	
p38 MAPK	NM_001353939.1	F: GCGAGTCCCTAATGCCTACG	199
		R: ACAACTGTTGAGCCACACTCA	
IL-1β	NM_204524.1	F: ACTGGGCATCAAGGGCTACA	142
		R: GCTGTCCAGGCGGTAGAAGA	
PGC-1ɑ	XM_015285697.2	F: CCAAAGGACACGCTCTAGATCA	76
		R: TCTCGATCGGGAATATGGAGAA	
Nrf2	XM_025152148.1	F: ATCACGAGCCCTGAAACCAA	143
		R: GGCTGCAAAATGCTGGAAAA	
Keap1	XM_025145847.1	F: GTACCAGATCGACAGCGTGG	197
		R: GGCAGTGGGACAGGTTGAAG	

JNK, c-Jun N-terminal kinase; TNF-ɑ, tumor necrosis factor-ɑ; IL, interleukin; p38 MAPK, p38 mitogen-activated protein kinase; PGC-1ɑ, peroxisome proliferator-activated receptor γ coactive-tor-1ɑ; Nrf2, Nuclear factor erythroid 2-related factor 2; Keap1, kelch like ECH associated protein 1.

### 2.11 Statistical analysis

SPSS 22.0 statistical software (SPSS Inc., Chicago, IL, United States) was used for general linear model (univariate) analysis. The Duncan’s multiple comparison method was employed for significant difference analysis, with *p* < 0.05 serving as the criterion for significant difference, and *p* < 0.01 indicating extremely significant difference. All results were expressed as mean ± standard deviation (SD).

## 3 Results

### 3.1 Growth performance

The effects of dietary supplementation of MCC on growth performance are presented in Table 3. In the 1–28 d period, the F/G ratio was significantly lower (*p* < 0.05) in MCC75 group compared to the in the CON group, while no significant difference (*p* > 0.05) was observed in ADFI and ADG among the 4 groups. In the 29–56 d period, the MCC150 and CTC groups exhibited significantly higher ADG (*p* < 0.05) and lower F/G ratio (*p* < 0.05) compared to the CON group. There was no significantly difference (*p* > 0.05) observed in ADFI among the 4 groups. In the whole period of the experiment, the F/G ratio, the diarrhea rate, and the dead panning rate were lower (*p* < 0.05) in the CTC, MCC75, and MCC150 groups compared to the CON group. Additionally, compared to the CON group, both the MCC75 and CTC groups exhibited significantly greater ADG (*p* < 0.05).

**Table 3:** Effect of mulberry leaf flavonoids and carnosic acid complex (MCC) on growth performance of broilers.

Items		Groups				*p*-value
		CON	CTC	MCC75	MCC150	
d 1–28	ADFI (g/d)	45.75 ± 0.56	44.83 ± 0.56	44.35 ± 0.56	44.83 ± 0.56	0.333
	ADG (g/d)	19.20 ± 0.39	19.82 ± 0.39	20.30 ± 0.39	19.32 ± 0.45	0.215
	F/G	2.39 ± 0.05^a^	2.26 ± 0.05^ab^	2.19 ± 0.05^b^	2.31 ± 0.05^ab^	0.033
d 29–56	ADFI (g/d)	91.88 ± 1.55	90.72 ± 1.55	88.66 ± 1.55	87.60 ± 1.79	0.267
	ADG (g/d)	29.77 ± 0.55^b^	32.09 ± 0.55^a^	30.77 ± 0.55^ab^	31.68 ± 0.63^a^	0.032
	F/G	3.09 ± 0.07^a^	2.84 ± 0.07^b^	2.88 ± 0.07^ab^	2.77 ± 0.08^b^	0.027
d 1–56	Initial BW (g)	102.89 ± 1.07	100.98 ± 0.93	99.75 ± 0.93	100.98 ± 0.93	0.193
	Final BW (g)	1475.14 ± 17.08^b^	1554.46 ± 17.08^a^	1529.64 ± 17.08^ab^	1512.21 ± 17.08^ab^	0.021
	ADFI (g/d)	67.97 ± 0.78	67.69 ± 0.78	66.50 ± 0.78	66.03 ± 0.90	0.309
	ADG (g/d)	24.49 ± 0.30^b^	25.96 ± 0.30^a^	25.53 ± 0.30^a^	25.50 ± 0.34^ab^	0.012
	F/G	2.78 ± 0.05^a^	2.61 ± 0.05^b^	2.61 ± 0.05^b^	2.59 ± 0.05^b^	0.024
	Diarrhea rate (%)	7.84 ± 0.43^a^	2.16 ± 0.43^b^	1.65 ± 0.43^b^	0.77 ± 0.43^b^	<0.001
	Death rate (%)	9.38 ± 1.69^a^	1.56 ± 1.69^b^	1.56 ± 1.69^b^	1.56 ± 1.69^b^	<0.001

Data are presented as mean ± SD (*n* = 8). In the same row, values with no letter or the same letter superscripts mean no significant difference (*p* > 0.05), while with different small letter superscripts indicate a significant difference (*p* < 0.05). CON, basal diet; CTC, basal diet with 50 mg/kg chlortetracycline; MCC75, basal diet supplemented with 75 mg/kg MCC; MCC150, basal diet supplemented with 150 mg/kg MCC; BW, body weight; ADG, average daily gain; ADFI, average daily feed intake; F/G, feed-to-gain ratio.

### 3.2 Immune organ indexes

Table 4 showed the effect of MCC on the immune organ indexes of broilers. The spleen index of the MCC 75 group was markedly higher (*p* < 0.05) than that of the CON and CTC groups. However, there were no significant differences in the thymus index and bursa index among the 4 groups (*p* > 0.05).

**Table 4:** Effect of mulberry leaf flavonoids and carnosic acid complex (MCC) on indices immune organs of broilers (%).

Items	Groups				*p*-value
	CON	CTC	MCC75	MCC150	
Spleen index, %	0.17 ± 0.02^b^	0.17 ± 0.02^b^	0.23 ± 0.02^a^	0.20 ± 0.02^ab^	0.021
Thymus index, %	0.24 ± 0.02	0.23 ± 0.02	0.18 ± 0.02	0.25 ± 0.02	0.136
Bursa index, %	0.21 ± 0.02	0.22 ± 0.02	0.18 ± 0.02	0.18 ± 0.02	0.553

Data are presented as mean ± SD (*n* = 8). In the same row, values with no letter or the same letter superscripts mean no significant difference (*p* > 0.05), while with different small letter superscripts indicate a significant difference (*p* < 0.05). CON, basal diet; CTC, basal diet with 50 mg/kg chlortetracycline; MCC75, basal diet supplemented with 75 mg/kg MCC; MCC150, basal diet supplemented with 150 mg/kg MCC.

### 3.3 Apparent digestibility

The apparent digestibility of dry matter (DM) was significantly higher (*p* < 0.05) in the MCC150 group than in the CON group (Table 5). Furthermore, ether extract (EE) digestibility markedly improved by 7.66, 7.19, and 6.66% (*p* < 0.05) in the CTC, MCC75, and MCC150 groups, respectively, compared to the CON group. Additionally, the content of crude protein (CP) was significantly greater (*p* < 0.05) in the MCC75 and MCC150 groups compared to the CON and CTC groups, while no significant difference was observed among crude fiber (CF) and ash digestibility (*p* > 0.05).

**Table 5:** Effect of mulberry leaf flavonoids and carnosic acid complex (MCC) on nutrient apparent digestibility of broilers (%).

Items	Groups				*p*-value
	CON	CTC	MCC75	MCC150	
DM	94.82 ± 0.24^b^	95.25 ± 0.24^ab^	95.52 ± 0.26^ab^	95.95 ± 0.24^a^	0.019
CP	72.45 ± 1.28^b^	73.28 ± 1.28^b^	77.27 ± 1.28^a^	77.41 ± 1.28^a^	0.019
EE	82.15 ± 1.46^b^	88.44 ± 1.46^a^	88.06 ± 1.46^a^	87.62 ± 1.46^a^	0.015
CF	39.99 ± 4.51	38.64 ± 4.03	40.32 ± 4.03	43.62 ± 4.51	0.870
Ash	22.18 ± 2.23	25.23 ± 1.93	26.80 ± 1.93	24.80 ± 2.07	0.491

Data are presented as mean ± SD (*n* = 8). In the same row, values with no letter or the same letter superscripts mean no significant difference (*p* > 0.05), while with different small letter superscripts indicate a significant difference (*p* < 0.05). CON, basal diet; CTC, basal diet with 50 mg/kg chlortetracycline; MCC75, basal diet supplemented with 75 mg/kg MCC; MCC150, basal diet supplemented with 150 mg/kg MCC; DM, dry matter; CP, crude protein; EE, ether extract; CF, crude fiber.

### 3.4 Intestinal digestive enzyme activity

The digestive enzyme activity of amylase, lipase, and trypsin in the jejunum of broilers was presented in Table 6. Compared with the control group, the amylase activity in the jejunum was significantly increased in the MCC and CTC groups (*p* < 0.05). However, no significantly differences were observed in the activities of jejunal lipase and trypsin among the 4 groups (*p* > 0.05).

**Table 6:** Effect of mulberry leaf flavonoids and carnosic acid complex (MCC) on digestive enzyme activity of jejunum in broilers.

Items	Groups				*p*-value
	CON	CTC	MCC75	MCC150	
α-Amylase (U/mgprot)	170.34 ± 96.05^c^	766.99 ± 96.05^a^	394.85 ± 88.93^bc^	538.28 ± 83.18^ab^	0.005
Lipase (U/mgprot)	244.52 ± 97.45	622.76 ± 97.45	362.44 ± 97.45	440.84 ± 97.45	0.071
Trypsin (U/mgprot)	19173.67 ± 8230.13	29111.33 ± 7361.25	18689.53 ± 8230.13	19395.61 ± 7361.25	0.366

Data are presented as mean ± SD (*n* = 8). In the same row, values with no letter or the same letter superscripts mean no significant difference (*p* > 0.05), while with different small letter superscripts (a, b, and c) indicate a significant difference (*p* < 0.05). CON, basal diet; CTC, basal diet with 50 mg/kg chlortetracycline; MCC75, basal diet supplemented with 75 mg/kg MCC; MCC150, basal diet supplemented with 150 mg/kg MCC.

### 3.5 Histomorphology of intestinal tract

**Figure 1:**
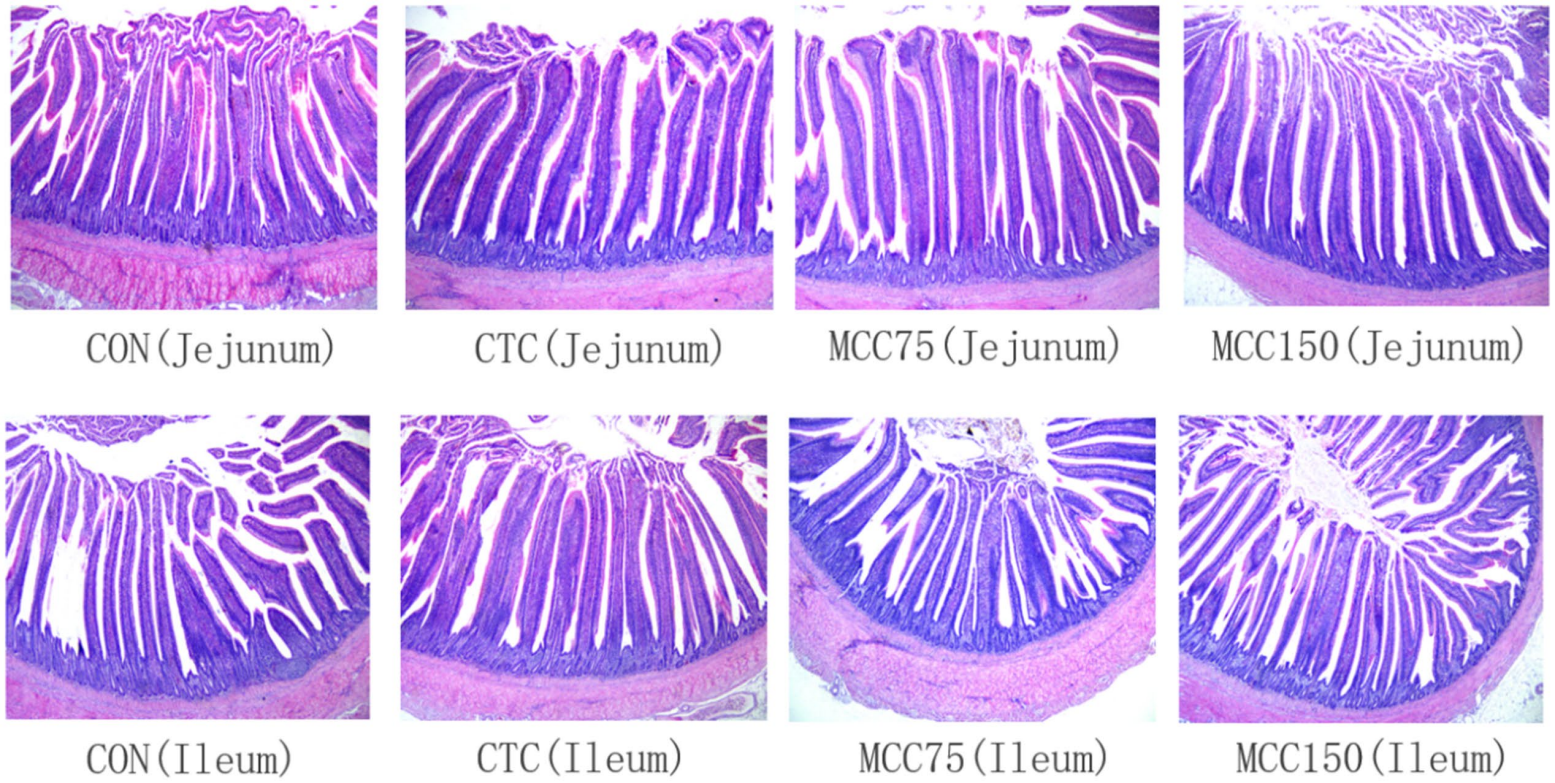
Morphological structure of jejunum and ileum in yellow-feathered broilers (HE staining, 40×). CON, basal diet; CTC, basal diet with 50 mg/kg chlortetracycline; MCC75, basal diet supplemented with 75 mg/kg MCC; MCC150, basal diet supplemented with 150 mg/kg MCC.

Dietary MCC150 decreased the crypt depth (*p* < 0.05) and increased the V/C ratio value (*p* < 0.05) in the jejunal (Table 7; Figure 1). In the ileal, the villus height and villus height-to-crypt depth (V/C) ratio value were increased (*p* < 0.01) in the CTC, MCC75, and MCC150 groups. Furthermore, the MCC150 group decreased the crypt depth in the ileal compared to the CON group (*p* < 0.05).

**Table 7:** Effect of mulberry leaf flavonoids and carnosic acid complex (MCC) on intestinal tissue morphology in broilers.

Items		Groups				*p*-value
		CON	CTC	MCC75	MCC150	
Jejunum	Villus height, μm	716.28 ± 40.52	784.16 ± 40.52	830.97 ± 36.99	814.21 ± 40.52	0.218
	Crypt depth, μm	139.04 ± 3.74^a^	134.45 ± 4.10^a^	138.28 ± 4.10^a^	122.45 ± 4.10^b^	0.035
	V/C ratio	5.15 ± 0.36^b^	5.82 ± 0.40^b^	6.14 ± 0.36^ab^	6.65 ± 0.40^a^	0.024
Ileum	Villus height, μm	496.42 ± 49.24^b^	749.69 ± 55.04^a^	696.46 ± 55.04^a^	707.37 ± 60.30^a^	0.006
	Crypt depth, μm	155.31 ± 3.12^a^	148.77 ± 3.12^ab^	146.2 ± 3.12^ab^	141.01 ± 3.12^b^	0.031
	V/C ratio	3.24 ± 0.31^b^	5.03 ± 0.25^a^	4.76 ± 0.25^a^	5.01 ± 0.28^a^	0.001

Data are presented as mean ± SD (*n* = 8). In the same row, values with no letter or the same letter superscripts mean no significant difference (*p* > 0.05), while with different small letter superscripts indicate a significant difference (*p* < 0.05). CON, basal diet; CTC, basal diet with 50 mg/kg chlortetracycline; MCC75, basal diet supplemented with 75 mg/kg MCC; MCC150, basal diet supplemented with 150 mg/kg MCC; V/C ratio, villus height-to-crypt depth (V/C) ratio.

### 3.6 Serum antioxidation

According to the data presented in Table 8, it can be observed that dietary supplementation with MCC75, MCC150, and CTC significantly increased the T-AOC values in serum (*p* < 0.01). Additionally, the CAT activities were significantly increased (*p* < 0.05) in the CTC and MCC150 groups compared to the CON group. It is worth noting that dietary supplementation with MCC150 also led to an increase in GSH-Px levels (*p* < 0.05). However, there were no significant differences (*p* > 0.05) in T-SOD and MDA levels among the 4 groups.

**Table 8:** Effect of mulberry leaf flavonoids and carnosic acid complex (MCC) on serum antioxidant indexes of broilers.

Items	Groups				*p*-value
	CON	CTC	MCC75	MCC150	
T-AOC (U/mL)	0.97 ± 0.11^c^	1.82 ± 0.11^a^	1.30 ± 0.10^b^	1.54 ± 0.10^ab^	<0.001
SOD (U/mL)	773.95 ± 45.99	866.15 ± 45.99	811.54 ± 65.03	692.42 ± 49.16	0.105
GSH-Px (U/mL)	1588.80 ± 41.30^b^	1709.68 ± 41.30^ab^	1611.58 ± 41.30^ab^	1729.74 ± 38.64^a^	0.049
CAT (U/mL)	13.69 ± 1.67^b^	20.21 ± 1.56^a^	17.92 ± 1.67^ab^	19.75 ± 1.67^a^	0.038
MDA (nmol/mL)	12.25 ± 1.40	12.49 ± 1.52	11.83 ± 1.52	12.52 ± 1.52	0.987

Data are presented as mean ± SD (*n* = 8). In the same row, values with no letter or the same letter superscripts mean no significant difference (*p* > 0.05), while with different small letter superscripts (a, b, and c) indicate a significant difference (*p* < 0.05). CON, basal diet; CTC, basal diet with 50 mg/kg chlortetracycline; MCC75, basal diet supplemented with 75 mg/kg MCC; MCC150, basal diet supplemented with 150 mg/kg MCC; T-AOC, total antioxidant capacity; SOD, superoxide dismutase; GSH-Px, glutathione peroxidase; CAT, catalase; MDA, malondialdehyde.

### 3.7 Expression of antioxidant related genes in intestinal p38-MAPK/Nrf2 pathway

**Figure 2:**
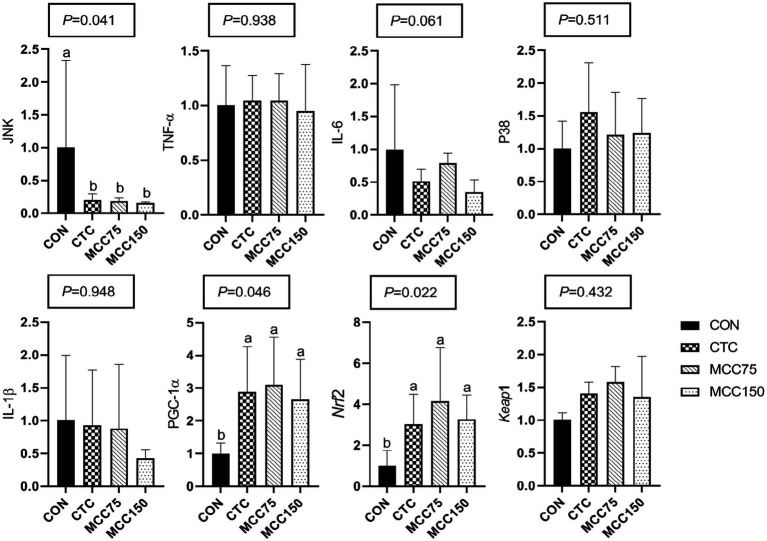
Effect of MCC on antioxidation-related gene expression in p38-MAPK/Nrf2 pathway in jejunum. Bars represent the means ± SD (*n* = 8), bars with different letters on top represent statistically significant results (*p* < 0.05). CON, basal diet; CTC, basal diet with 50 mg/kg chlortetracycline; MCC75, basal diet supplemented with 75 mg/kg MCC; MCC150, basal diet supplemented with 150 mg/kg MCC; JNK, c-Jun N-terminal kinase; TNF-ɑ, tumor necrosis factor-ɑ; IL, interleukin; p38 MAPK, p38 mitogen-activated protein kinase; PGC-1ɑ, peroxisome proliferator-activated receptor γ coactive-tor-1ɑ; Nrf2, nuclear respiratory factor 2; Keap1, kelch like ECH associated protein 1.

In the jejunum, dietary supplementation with CTC, MCC75, and MCC150 decreased (*p* < 0.05) the relative mRNA expression abundance of C-Jun N-terminal kinase (JNK), while increasing the mRNA levels of Peroxisome proliferator-activated receptorγcoactivetor-1ɑ (PGC-1) and nuclear factor erythroid 2-related factor 2 (Nrf2) (*p* < 0.05) (Figure 2).

**Figure 3:**
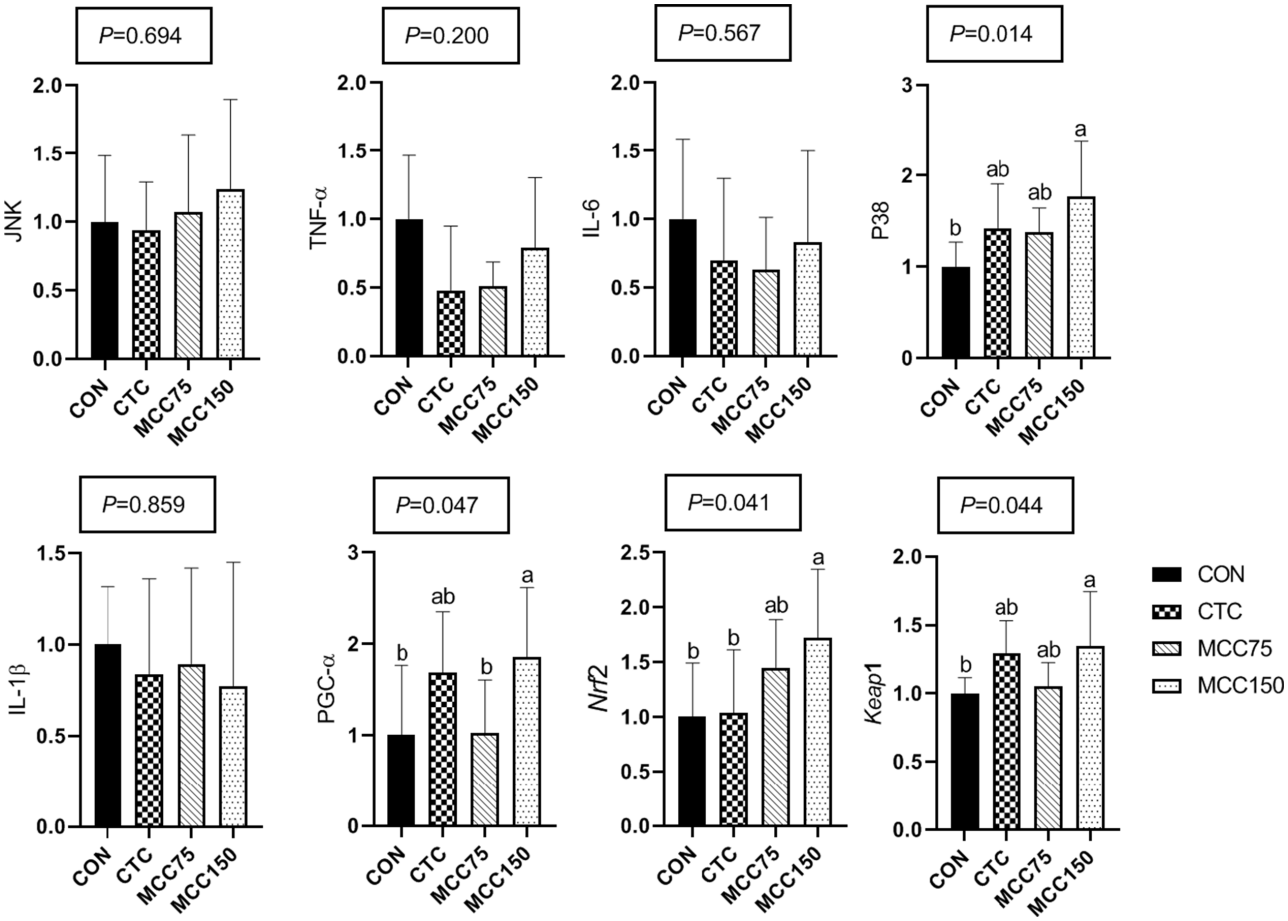
Effect of MCC on antioxidation-related gene expression in p38-MAPK/Nrf2 pathway in ileum. Bars represent the means ± SD (*n* = 8), bars with different letters on top represent statistically significant results (*p* < 0.05). CON, basal diet; CTC, basal diet with 50 mg/kg chlortetracycline; MCC75, basal diet supplemented with 75 mg/kg MCC; MCC150, basal diet supplemented with 150 mg/kg MCC; JNK, c-Jun N-terminal kinase; TNF-ɑ, tumor necrosis factor-ɑ; IL, interleukin; p38 MAPK, p38 mitogen-activated protein kinase; PGC-1ɑ, peroxisome proliferator-activated receptor γ coactive-tor-1ɑ; Nrf2, nuclear respiratory factor 2; Keap1, kelch like ECH associated protein 1.

In the ileum, dietary supplementation with MCC150 increased the relative mRNA expression abundance of P38 mitogen-activated protein kinase (P38), PGC-1, Nrf2, and Kelch like ECH associated protein 1 (Keap1) (*p* < 0.05) (Figure 3). Notably, the mRNA expression levels of Nrf2 were markedly higher (*p* < 0.05) in the MCC150 group than in the CTC group.

## 4 Discussion

Flavonoids, which are anti-inflammatory and antioxidant chemicals, can influence animal immunity and growth performance by regulating lipid metabolism, immunological function, and growth axis function (1, 7). Simultaneously, carnosic acid, a plant phenolic acid molecule, exhibits antibacterial, anti-inflammatory, antioxidant, hypoglycemic, and hypolipidemic properties (9, 10). It can function as an antibiotic replacement by modulating lipid metabolism and blocking cholinesterase, thereby improving animal growth (13). A previous study found that dietary supplementary with 200 mg/kg, 400 mg/kg, and 800 mg/kg of flavonoid (quercetin) could increase ADG in broilers (14). In the present study, the ADG increased and the F/G ratio, diarrhea rate, and dead panning rate decreased when supplemented with MCC or chlortetracycline, indicating that the synergistic effect of MCC could promote broiler growth and development with comparable antibiotic efficacy. Nutrient metabolism can influence animal growth, and it has been demonstrated that mulberry leaf flavonoids increase the rate of metabolism in calves after weaning (1). Moreover, because flavonoids have a structure similar to estradiol, they can interact with the hypothalamus and pituitary gland, helping to regulate hormone levels and promote growth in animals. Qi et al. (15) also revealed that *Allium* flavones could boost serum hormone and insulin-like growth factor-1 levels, thereby promoting broiler chicken growth. Therefore, the promotive effect of MCC on growth performance is likely closely associated with the presence of mulberry leaf flavonoids. These results suggest that supplementing an appropriate amount of MCC into the diets of broilers is feasible.

The immune organ index is commonly used to measure the immune system function and overall health status of animals. There is a positive correlation between the immunological organ index and immune organ development, and immune function rises with immune organ index (16). The thymus, spleen, and Fabricius bursa play crucial roles as immune organs in birds, and their indices serve as valuable indicators for assessing the organism’s immune status (8, 17). The thymus functions as a central immune organ that secretes T lymphocytes and also plays a significant role in the neuroendocrine network (8). The spleen, as an important peripheral immune organ, directly affects broiler immunity (6, 17). Furthermore, previous research has demonstrated that plant flavonoids can promote the development of immune organs and enhance animal immunity (8). For example, the supplementation of alfalfa flavonoids in the feed can enhance the growth performance, spleen and bursa weights, as well as aspartate transaminase activity of meat geese (8). The results of this experiment and the above conclusion show some similarities, as MCC was observed to increase the spleen index and facilitate the development of immune organs. This effect could potentially be attributed to the action of mulberry leaf flavonoids in increasing protein synthesis and secretion while fully promoting the immune mechanisms of the spleen.

Animal growth and development are related to apparent digestibility of nutrients, which can directly reflect the digest and absorb ability of the animal body (18). Recent studies have demonstrated the positive effects of rosemary extract on enhancing nutrient digestibility in weaned piglets (19). Additionally, supplementation of 1,000 mg/kg quercetin has been found to increase the apparent digestibility of DM and nitrogen in growing pigs (20). Moreover, flavonoids derived from mulberry leaves have shown the potential to improve the digestibility of DM, CF, and metabolizable energy in broilers (21). In the present study, our results showed that the MCC increased the apparent digestibility of DM, CP, and CF in broilers. This suggested that MCC could effectively improve the apparent digestibility of nutrients in broilers, with a similar effect to that of chlortetracycline. The rate of nutrient digestibility and absorption in animals has a favorable correlation with animal growth, which further supports the aforementioned finding that MCC could enhance broiler growth performance. Gut microbiota plays a crucial role in digestive processes and possess a diverse metabolic repertoire closely associated with food metabolism (22). Furthermore, a study has demonstrated that flavonoids and carnosic acid can induce changes in gut microbiota composition (23). Flavonoids can increase nutrient digestion and utilization by promoting the growth of probiotics in the intestine while inhibiting the growth of harmful bacteria (24). Therefore, it is hypothesized that the MCC could improve nutrient digestibility in broilers via influencing intestinal bacteria in broilers. Unfortunately, the intestinal bacteria were not detected in our study. Mulberry leaf flavonoids could also improve nutritional digestion and absorption in broilers by boosting intestinal villus formation, expanding the area of intestinal digestion and absorption, and enhancing the activity and production of intestinal digestive enzymes (25).

The enzymes in the intestine of chickens, namely amylase, lipase, and trypsin, play a vital role in the digestion and breakdown of nutrients. Flavonoids have been proven to increase the activity of digestive enzymes. Ding et al. (1) observed that mulberry leaf flavonoids might enhance the growth of intestinal villi in broilers and dramatically increase digestive enzyme activity. Figueroa-Perez et al. (26) demonstrated that flavonoids could boost the growth of good bacteria in the colon, limit the proliferation of dangerous bacteria, and enhance intestinal trypsin and amylase activity. Moreover, relevant studies have demonstrated that MCC could affect the composition and activity of the microbiota, improving the growth of intestinal epithelial cells and exerting beneficial effects on intestinal barrier function and gastrointestinal inflammation (27, 28). Our results showed that combining chlortetracycline with 150 mg/kg MCC could significantly increase the activity of jejunum-amylase in broilers, indicating that MCC could increase the activity of intestinal digestive enzymes in broilers. This may be related to the modulation of microbial metabolism in the intestine by MCC, thereby promoting the secretion of digestive enzymes and enhancing the activity of relevant digestive enzymes (28).

The small intestine is the primary site of nutritional absorption in animals, and the morphological structure of the gut plays an essential role in the digestion and absorption of numerous nutrients (29). Under normal conditions, intestinal villi can significantly increase the surface area for nutrients digestion and absorption. Additionally, the crypt depth is inversely correlated with the ability of intestinal epithelial cells to secrete digestive juices, whereas a larger V/C ratio corresponds to a higher digestive and absorptive capacity in the intestine (30). By enhancing the alkaline phosphatase activity of intestinal epithelial cells, hawthorn flavone compounds can encourage epithelial cell growth and proliferation, enhance the intestinal epithelial barrier, and promote the development of intestinal villous tissue (31). Flavonoids have been shown to increase the height of the ileal villus in broilers (32). Additionally, carnosic acid improved intestinal crypt architecture and goblet cell loss, according to research conducted by Yang et al. (33). In mice with colitis, rosemary extract supplemented with carnosic acid was able to enhance intestinal barrier integrity (28). In this study, 150 mg/kg MCC could significantly increase the villus height and V/C value of the jejunum and ileum while decreasing the crypt depth in broilers. These results were in agreement with the previous research (34). Broilers’ intestinal tracts may benefit from the MCC because flavonoids lower oxidative stress by inhibiting inflammatory factors, down-regulating the expression of NADPH oxidase, and up-regulating the intestinal hormone glucagon-like peptide (GLP)-2, which strengthens the intestinal barrier (34). As a result, the MCC complex can promote the secretion of jejunal amylase, the development of intestinal villi, and effectively improve the morphological structure of the intestinal tract. This leads to a greatly increase in the digestion and absorption area of nutrients in the intestinal tract, and improves the apparent digestibility of nutrients in broilers, thereby improving broiler growth performance.

The strength of antioxidant performance is a key indicator of physical health, reflecting the level of the body’s antioxidant defense system and its ability to scavenge free radicals (35). SOD plays a vital role in scavenging superoxide anion radicals in the body and maintaining a balance between oxidation and anti-oxidation process (36). GSH-Px, as an essential peroxidase in the body, catalyzes the conversion of GSH into oxidized glutathione and efficiently eliminating hydrogen peroxide (37). SOD and GSH-Px can effectively eliminate excessive free radicals and prevent peroxides from damaging the structure and function of the cell membrane. Additionally, CAT decomposes hydrogen peroxide in the body and acts as an important antioxidant enzyme (36). The MDA is one of the byproducts of lipid peroxide metabolism in the body, created by the action of oxygen free radicals on the membrane. The MDA level is inversely connected with the level of cellular oxidative damage and can serve as an indirect indicator (38). Carnosic acid dramatically enhanced the levels of GSH and SOD, and decreased the level of MDA caused by DSS, according to Yang et al. (33); this indicates that carnosic acid could be important in the development of treatments for inflammatory disorders linked to oxidative stress. By controlling prooxidants and antioxidant enzymes, carnosic acid can increase broilers’ antioxidant capacity (39). It has been demonstrated that the bioavailability of flavonoids could limited when supplemented in animal diets and may be less likely to directly exert antioxidant capacity (40). The mulberry leaf flavonoids are able to incorporate substantial antioxidant activity by scavenging free radicals and chelating metals (41). The antioxidant capacity increased and plasma MDA levels decreased when dietary supplementation of total flavonoids from *Artemisia annua* in Wenchang hens, as shown in a study by Guo et al. (7). Similarly, alfalfa flavonoids were found to enhance plasma T-AOC activity as well as the gene expression of antioxidant enzymes in broilers (42). Chen et al. (43) also demonstrated that the flavonoid quercetin could alleviate changes in CAT and SOD activities in oxidatively injured cells. In the present study, our results showed that MCC and chlortetracycline could increase serum T-AOC levels and the activities of CAT and GSH-Px, indicating that MCC could effectively increase the antioxidant level of broilers, which was in agreement with a previous study in broilers (42).

To elucidate how MCC might enhance the antioxidant capacity of broilers, we examined the role of the p38 mitogen-activated protein kinase (p38 MAPK) signaling pathway. The p38 MAPK signaling pathway is a common mechanism for intracellular information transmission, primarily involved in gene transcription, stress response, inflammatory response, and cellular immune regulation (44). The p38 MAPK pathway is critical in regulating the expression of several antioxidant enzyme genes (45). Moreover, the p38 signaling pathway stimulates the production of the transcription factor Nrf2 (46), which further enhances the cellular antioxidant defense system. Peroxisome proliferator receptor gamma coactivator 1 (PGC-1) is a transcriptional regulator that plays an important function in the anti-oxidative stress system and can enhance the body’s antioxidant capacity by promoting the production of cellular antioxidant enzymes (47). The Nrf2-Keap1 signaling pathway is one of critical pathway for cellular protection against oxidative stress. Under normal physiological conditions, Nrf2 binds to Keap1, forming a complex that is recognized and degraded by the proteasome via polyubiquitinated markers. However, when the Nrf2-Keap1 pathway is activated, the complex dissociates, allowing Nrf2 to translocate into the nucleus. Once in the nucleus, Nrf2 binds to the antioxidant response element (ARE) and stimulates the transcription of genes involved in antioxidant enzymes, thereby enhancing cellular antioxidant capacity and tolerance to oxidative stress (48). Our results revealed that MCC and chlortetracycline could reduce JNK mRNA expression while increasing PGC-1 and Nrf2 mRNA expression in broiler jejunal mucosa. The combination of 150 mg/kg MCC increased the expression of p38, PGC-1, Nrf2, and Keap1 mRNA in broiler ileal mucosa. Flavonoids containing carnosic acid have been shown to possess robust antioxidant properties by activating the Nrf2-Keap1 pathway. Previous studies have reported that flavonoids facilitate the transcription of Nrf2 to the nucleus, where it binds to antioxidant response element (ARE) and stimulates the transcription of antioxidant proteins, phase II detoxifying enzymes, and other genes (49). Flavonoids may also interact with AhR (aryl hydrocarbon receptor), leading to the dissociation of the Keap1/Nrf2 complex and facilitating Nrf2 translocation into the nucleus, thereby enhancing the transcription of antioxidant enzymes such as superoxide dismutase, glutathione peroxidase, and catalase (50). Furthermore, Lee and Jang (51) demonstrated that carnosic acid could promote Nrf2 nuclear displacement, effectively reducing the generation of harmful ROS (reactive oxygen species) and promoting the translation of phase II antioxidant enzymes. Carnosic acid may also protect against DSS-induced decreases in Nrf2 protein levels by interfering with the interaction of Cullin3 and Keap1 (33). The results of this study were basically consistent with the above studies, suggesting that MCC could influence gene expression in the intestinal p38-MAPK/Nrf2 signaling pathway and improve the ability of broilers to resist oxidative stress, and thus promote growth. However, further investigations are needed to elucidate the underlying mechanisms of p38-MAPK/Nrf2 activation by MCC. Furthermore, it is widely recognized that antioxidants play a pivotal role in protecting against inflammatory diseases, as oxidative stress and inflammation are intricately interconnected (52, 53, 54). In light of this, it is plausible to hypothesize that MCC may also contribute to reducing inflammation in broilers, thus warranting further exploration.

## 5 Conclusion

Collectively, these results demonstrate that dietary supplementation with MCC could effectively improve growth performance, intestinal morphology, nutrient absorption, and antioxidant capacity in broilers, which may be related to regulation of the MAPK/Nrf2 signaling pathway. These findings provide valuable insights into the potential benefits of MCC for broiler performance. Thus, it is feasible and beneficial to use MCC at a dosage of 150 mg/kg as an antibiotic alternative in the diet of broilers. Future research will focus on elucidating the mechanisms of MCC’s effects on the MAPK/Nrf2 pathway and assessing its long-term impacts and economic feasibility in commercial broiler production.

## Data availability statement

The original contributions presented in the study are included in the article/Supplementary material, further inquiries can be directed to the corresponding authors.

## Ethics statement

The animal study was approved and conducted in strict accordance with the guidelines recommended and ethically approved by the Institutional Animal Care and Use Committee of Hunan Agricultural University (Permit Number: CACAHU 2020-0821). The study was conducted in accordance with local legislation and institutional requirements.

## Author contributions

CL: Investigation, Writing – original draft. HH: Data curation, Methodology, Validation, Writing – original draft. YC: Supervision, Writing – original draft, Writing – review & editing. YZ: Investigation, Writing – review & editing. TM: Methodology, Writing – review & editing, Software. BT: Investigation, Writing – review & editing. WH: Conceptualization, Formal analysis, Methodology, Writing – review & editing. XF: Data curation, Formal analysis, Methodology, Writing – review & editing. DX: Funding acquisition, Methodology, Project administration, Supervision, Validation, Writing – review & editing.
